# Stratified risks of infection-related hospitalization in patients with chronic kidney disease - A prospective cohort study

**DOI:** 10.1038/s41598-020-61144-6

**Published:** 2020-03-11

**Authors:** Wei-Shun Yang, Yi-Cheng Chang, Meng-Lun Hsieh, Jiun-Ling Wang, Li-Chiu Wu, Chia-Hsuin Chang

**Affiliations:** 1 National Taiwan University Hospital, Hsin-Chu Branch, Department of Internal Medicine, Hsin-Chu City, Taiwan; 2 National Taiwan University, College of Medicine, Taipei, Taiwan; 3 National Taiwan University, The Graduate Institute of Medical Genomics and Proteomics, Taipei, Taiwan; 4 National Taiwan University Hospital, Department of Internal Medicine, Taipei, Taiwan; 5 Academia Sinica, Institute of Biomedical Sciences, Taipei, Taiwan; 6 Department of Internal Medicine, National Cheng Kung University Hospital, Tainan, Taiwan; 7 College of Medicine, National Cheng Kung University, Tainan, Taiwan

**Keywords:** Bacterial infection, Chronic kidney disease, Epidemiology

## Abstract

Patients with chronic kidney disease (CKD) are at high risk of infection, but whether the risks are attenuated in different patient groups remains unclear. This study enrolled participants with CKD stages 1–3 in the New Taipei City Health Screening Program between 2005 and 2008. A proportional hazard regression model was employed to calculate the hazard ratios (HRs) and 95% confidence intervals (CIs) for infection-related hospitalization and mortality in younger (<50-year-old) and older (≥50-year-old) CKD patients. Of 119,871 adults, there were 14,207 cases of first hospitalization for infection during a median follow-up of 8.14 years; 45.5% of these cases were younger patients. Unlike CKD stage 1 and 2 patients, the risk of infection-related hospitalization in younger CKD stage 3 patients is as high as for older CKD stage 3 patients. Proteinuria increases the risk of infection-related hospitalization independent of estimated glomerular filtration rate (eGFR) levels in older CKD patients but this relationship is weak in their younger counterparts. In conclusion, the risk of infection-related hospitalization is high in subgroups of CKD patients. Prevention and treatment of infections in these patients merit more attention.

## Introduction

The burden of chronic kidney disease (CKD) is increasing worldwide, with a mean prevalence of 11 to 13%^1,2^. Advanced CKD and end-stage renal disease (ESRD) are associated with high mortality and morbidity^3–5^. Even a mild decline in glomerular filtration rate poses a threat to a patient’s wellbeing, as CKD stage 1 or 2 patients already have higher mortality and hospitalization rates than the non-CKD population^6^, with infection being one of the leading causes. Furthermore, CKD patients hospitalized for infection have greater risks of treatment- or disease-related complications, intensive care unit admission, longer length of stay, and higher total cost than non-CKD patients^7–13^. However, unlike the much emphasized prevention of cardiovascular disease in the early CKD population, there is still a need of better prevention against infection in these patients^14^.

Previous studies demonstrated that not only advanced CKD or ESRD patients but also those with moderately impaired renal function are threatened by a higher infection risk^15–21^. However, previous studies enrolled mostly limited patient groups or focused on limited types of infection. For example, studies enrolling patients older than 65 years concluded that the risks of infection were not modified by age^15,19^, but James *et al*. noted that in a population with broader age range, the risk of pneumonia was more prominent in younger CKD patients, 18 to 54 years of age^18^. Whether other types of infection is also influenced by age is unclear. Moreover, two studies demonstrated that proteinuria is a risk factor for infection in CKD patients, independent of eGFR levels, but one study was limited to diabetes patients^19^ and the other focused on only four most common infections (sepsis, pneumonia, urinary tract and cellulitis)^17^. Again, the relationship beween proteinuria and infection in younger and older CKD patients is unclear. All the aforementioned studies were conducted in western countries and the risks in different countries or ethnicities remains to be clarified. This research is a large Asian community-based, prospective cohort study on the relationship between eGFR and the incidences of hospitalization for all infections, site-specific infections, and infection-related mortality across the whole spectrum of renal functions and age groups.

## Results

After excluding individuals who did not meet inclusion criteria, 119,871 out of 125,865 participants in the New Taipei City Community Health Screening Program between 2005 and 2008 (mean age of 51.51 years; 35.7% men) were included in the analysis (Supplementary Fig. 1). Among them, 23.4% had CKD stage 1 high (eGFR > 105 ml/min/1.73 m^2^); 33.8%, CKD stage 1 (eGFR 90–105 ml/min/1.73 m^2^); 38.1%, CKD stage 2 (eGFR 60–89 ml/min/1.73 m^2^); and 4.8%, CKD stage 3 (eGFR 30–59 ml/min/1.73 m^2^). Compared with stage 1 subjects, participants with lower eGFR were more likely to be men and older, overweight or obese, and have a lower education level (Table 1). They were also more likely to have comorbidities including diabetes, hypertension, cardiovascular disease, cerebrovascular disease, peripheral vascular disease, dyslipidemia, chronic lung disease, and cancer. The aforementioned comorbidities plus obesity were more prevalent among older patients, but their younger counterparts tend to smoke or consume alcohol more often (Table 2). Owing to the optional nature of urinanalysis in the health screening program, only 69.04% of the younger patients had data regarding proteinuria, in contrast to 98.68% of the older patients (Table 2).

**Table 1:** Characteristics of study participants in different chronic kidney disease stages at study entry (N = 119.871).

CKD stages	Stage 1, high	Stage 1	Stage 2	Stage 3
eGFR, ml/min/1.73 m^2^	>105	90–105	60–89	30–59
Number	28,013	40,502	45,665	5,691
Male (%)	18.54	31.57	47.31	55.82
Age < 50 (%)	93.69	44.52	30.93	3.67
**BMI (%)**				
Underweight	4.91	2.37	1.99	2.42
Normal	66.78	58.94	53.41	46.34
Overweight or obese	28.31	38.69	44.59	51.24
Current cigarette smoker (%)	12.27	14.07	17.08	16.53
Non-abstainer (%)	38.47	37.89	38.16	25.27
**Education level (%)**				
Illiterate/did not attend school	1.44	7.67	14.11	30.84
Elementary school	10.06	28.05	29.66	36.27
Junior high and school	58.42	44.75	38.67	24.54
College and graduate school	30.09	19.53	17.55	8.34
**Comorbidities, %**				
Diabetes	4.43	8.62	9.79	21.52
Hypertension	5.10	14.84	24.37	55.65
Ischemic heart disease	1.27	4.31	8.10	20.31
Myocardial infarction	0.05	0.11	0.31	1.04
Cardiac dysrhythmia/atrial fibrillation	1.28	2.09	3.39	7.68
Congestive heart failure	0.29	0.85	1.61	5.89
Stroke	0.23	0.77	1.60	5.31
Peripheral vascular disease	0.11	0.42	0.53	1.05
Disorders of lipid metabolism	4.60	11.35	14.30	25.65
Chronic lung disease	4.32	6.31	9.54	17.71
Chronic liver disease	5.53	7.03	7.89	8.65
Autoimmune disease	2.30	2.68	2.82	3.46
Dementia	*	0.05	0.32	1.56
Cancer	0.95	1.68	2.32	4.41
Charlson comorbidity score, mean (SD)	0.22 (0.60)	0.37 (0.80)	0.50 (0.92)	1.08 (1.39)
**Laboratory data**				
Albumin in g/dL, mean (SD)	4.62 (0.26)	4.61 (0.25)	4.60 (0.26)	4.51 (0.30)
Dipstick proteinuria (%)**				
−	91.74	90.48	87.26	72.66
+/−	7.02	8.05	10.26	16.64
1+	0.89	1.03	1.75	6.36
≥2+	0.34	0.43	0.73	4.34
**Drug use and hospitalization history, %**				
Systemic steroid use >30 days before study entry	0.69	1.07	1.55	3.27
Hospitalization within 6 months before enrollment	2.47	2.41	2.94	6.47
Hospitalization within 6 months before index hospitalization for infection	0.87	1.60	3.12	9.58

*The exact number of cases in both categories were too small to be retrieved because of the authority’s policy regulation.

**Number of cases with dipstick proteinuria test: 17,495 (62.45%) in eGFR > 105 ml/min/1.73 m^2^, 36,012 (88.92%) in eGFR 90–105 ml/min/1.73 m^2^, 43,107 (94.39%) in eGFR 60–89 ml/min/1.73 m^2^, 5,504 (96.72%) in eGFR 30–59 ml/min/1.73 m^2^.

**Table 2:** Characteristics of study participants stratified by age at study entry (N = 119,871).

Variable	<50 y/o (n = 54,535)	≥50 y/o (n = 65,336)
	Age	
Age, mean (SD)	41.43 (5.25)	60.39 (8.48)
Male (%)	33.72	37.30
**CKD stages**		
Stage 1 high	46.69	3.90
Stage 1	30.24	36.75
Stage 2	22.75	50.90
Stage 3	0.32	8.44
**BMI (%)**		
Underweight	3.93	1.89
Normal	63.49	53.54
Overweight and obese	32.58	44.57
Current smoker (%)	17.12	13.07
Non-abstainer (%)	45.04	31.26
**Comorbidities, %**		
Diabetes	3.22	13.27
Hypertension	5.41	28.75
Ischemic heart disease	1.35	9.52
Myocardial infarction	0.04	0.36
Cardiac dysrhythmia/atrial fibrillation	1.21	3.87
Congestive heart failure	0.31	2.03
Stroke	0.20	1.99
Peripheral vascular disease	0.11	0.68
Disorders of lipid metabolism	4.86	17.17
Chronic lung disease	4.17	10.49
Chronic liver disease	5.88	8.08
Autoimmune disease	2.23	3.06
Dementia	0.01	0.39
Cancer	0.92	2.69
Charlson comorbidity score, mean (SD)	0.21 (0.58)	0.59 (1.01)
**Dipstick proteinuria (%)**		
− to +/−	68.14	95.75
1+	0.64	1.97
≥2+	0.26	0.96
Not available	30.96	1.32
**Drug use and hospitalization history, %**		
Systemic steroid use >30 days before study entry	0.74	1.72
Hospitalization within 6 months before enrollment	2.17	3.36
Hospitalization within 6 months before index hospitalization for infection	0.85	3.67
Hospital stay (days)	7.90 (17.18)	11.83 (18.23)

### Hospitalization for infection

A total of 14,385 cases of first hospitalization for any infection occurred during a median follow-up of 8.14 years. The crude incidence of any hospitalization for infection per 1,000 person-years was 9.47 (95% CI: 9.07–9.88), 11.63 (95% CI: 11.26–12.02), 18.65 (95% CI: 18.20–19.11) and 55.67 (95% CI: 53.32–58.12) for subjects with CKD stage 1 high, stages 1, 2 and 3, respectively. In the Cox regression analysis, the crude HR of any hospitalized infection was 0.81 (95% CI: 0.77~0.86), 1.61 (95% CI: 1.54–1.67) and 4.84 (95% CI: 4.58–5.11) for subjects with CKD stage 1 high, CKD stages 2 and 3, respectively, when compared with CKD stage 1 subjects.

### Hospitalization for infection in younger (<50) and older (≥50) patients

The site-specific and age-stratified incidence of infection-related hospitalization and mortality are displayed in Table 3. The relationship between eGFR levels and infection-associated hospitalization was significantly modified by age (*p* value for test for interaction <0.001). Younger subjects with CKD stages 1 and 2 had lower aHR for infection-related hospitalization than their older counterparts, but the aHR was equally high for both younger and older CKD stage 3 patients (Table 4). The younger stage 3 CKD also had longer hospital stay (mean 23.33 days).

**Table 3:** Follow-up duration, number of incident cases, crude incidence of hospitalization for infection syndrome and infection-related mortality, and association between different eGFR categories and risk of hospitalization for infection syndrome and infection-related mortality as compared with eGFR 90–105 and younger patients (N = 119,871).

		No. of cases		Person-years		Incidence rate per 1,000 person-years	
Any infection	CKD stage	<50	≥50	<50	≥50	<50	≥50
	Stage 1 high	1,800	282	200,478	19,471	8.98 (8.57–9.40)	14.48 (12.89–16.28)
	Stage 1	1,078	2,564	128,575	184,461	8.38 (7.90–8.90)	13.90 (13.37–14.45)
	Stage 2	780	5,638	96,666	247,427	8.07 (7.52–8.66)	22.79 (22.20–23.39)
	Stage 3	35	2,030	1,266	35,830	27.66 (19.86–38.52)	56.66 (54.24–59.18)
Septicemia		<50	≥50	<50	≥50	<50	≥50
	Stage 1 high	159	53	200,478	19,471	0.79 (0.68–0.93)	2.72 (2.08–3.56)
	Stage 1	133	476	128,575	184,461	1.03 (0.87–1.23)	2.58 (2.36–2.82)
	Stage 2	101	1,139	96,666	247,427	1.04 (0.86–1.27)	4.60 (4.34–4.88)
	Stage 3	7	448	1,266	35,830	5.53 (2.64–11.60)	12.50 (11.40–13.72)
Lower respiratory tract infection^+^		<50	≥50	<50	≥50	<50	≥50
	Stage 1 high	241	66	200,478	19,471	1.20 (1.06–1.36)	3.39 (2.66–4.31)
	Stage 1	171	655	128,575	184,461	1.33 (1.14–1.55)	3.55 (3.29–3.83)
	Stage 2	117	1,944	96,666	247,427	1.21 (1.01–1.45)	7.86 (7.52–8.21)
	Stage 3	8	791	1,266	35,830	6.32 (3.16–12.64)	22.08 (20.5–23.67)
Intra-abdominal infection		<50	≥50	<50	≥50	<50	≥50
	Stage 1 high	316	43	200,478	19,471	1.58 (1.41–1.76)	2.21 (1.64–2.98)
	Stage 1	229	341	128,575	184,461	1.78 (1.56–2.03)	1.85 (1.66–2.06)
	Stage 2	156	619	96,666	247,427	1.61 (1.38–1.89)	2.50 (2.31–2.71)
	Stage 3	3	154	1,266	35,830	2.37 (0.76–7.35)	4.30 (3.67–5.03)
Reproductive and urinary tract infection		<50	≥50	<50	≥50	<50	≥50
	Stage 1 high	990	133	200,478	19,471	4.94 (4.64–5.26)	6.83 (5.76–8.10)
	Stage 1	487	1,020	128,575	184,461	3.79 (3.47–4.14)	5.53 (5.20~–5.88)
	Stage 2	346	2,068	96,666	247,427	3.58 (3.22–3.98)	8.36 (8.01–8.73)
	Stage 3	20	773	1,266	35,830	15.80 (10.20–24.50)	21.57 (20.11–23.15)
Skin and soft tissue infection		<50	≥50	<50	≥50	<50	≥50
	Stage 1 high	210	31	200,478	19,471	1.05 (0.91–1.20)	1.59 (1.12–2.26)
	Stage 1	129	391	128,575	184,461	1.00 (0.84–1.19)	2.12 (1.92–2.34)
	Stage 2	121	737	96,666	247,427	1.25 (1.05–1.50)	2.98 (2.77–3.20)
	Stage 3	3	256	1,266	35,830	2.37 (0.76–7.35)	7.14 (6.32–8.08)
Mortality from any infection		<50	≥50	<50	≥50	<50	≥50
	Stage 1 high	10*	4	20,7592	20,571	0.02 (0.01–0.04)*	0.19 (0.07–0.52)
	Stage 1		33	132,727	193,773		0.17 (0.12–0.24)
	Stage 2		222	99,643	265,487		0.84 (0.73–0.95)
	Stage 3		123	1,395	41,784		2.94 (2.47–3.51)

*The exact number of cases in both categories were too small to be retrieved because of the authority’s policy regulation. ^+^Including influenza, bacterial and viral pneumonia, bronchopneumonia and empyema.

**Table 4:** Adjusted hazard ratios* for patients of different CKD stages and risk of hospitalization for infection syndrome and infection-related mortality as compared with eGFR 90–105 and aged <50 years (N = 119,871).

		Stage 1 high		Stage 1		Stage 2		Stage 3	
		Age < 50 years (n = 26,245)	Age ≥ 50 years (n = 1,768)	Age < 50 years (n = 16,491)	Age ≥ 50 years (n = 24,011)	Age < 50 years (n = 14,124)	Age ≥ 50 years (n = 31,541)	Age < 50 years (n = 209)	Age ≥ 50 years (n = 5,482)
**Hospitalization for infection**									
All infections	Crude HR	1.07 (0.99–1.15)	1.73 (1.52–1.97)	Reference	1.66 (1.54–1.78)	0.96 (0.88–1.06)	2.72 (2.55–2.91)	3.32 (2.37–4.64)	6.84 (6.35~–7.36)
	Adjusted HR	1.11 (1.03–1.20)	1.42 (1.25–1.62)	Reference	1.23 (1.14~–1.33)	0.95 (0.87–1.04)	1.70 (1.58–1.82)	2.58 (1.84–3.61)	2.83 (2.61–3.07)
Septicemia and bacteremia	Crude HR	0.76 (0.61–0.96)	2.64 (1.92–3.63)	Reference	2.50 (2.06–3.03)	1.01 (0.78–1.31)	4.47 (3.74–5.35)	5.40 (2.53–11.55)	12.38 (10.20–15.03)
	Adjusted HR	0.82 (0.65–1.03)	2.10 (1.52–2.90)	Reference	1.73 (1.42–2.11)	0.99 (0.76~–1.28)	2.45 (2.03–2.96)	4.03 (1.88–8.62)	4.14 (3.36–5.10)
Lower respiratory tract^+^	Crude HR	0.90 (0.74–1.10)	2.55 (1.92~–3.39)	Reference	2.67 (2.26–3.16)	0.91 (0.72–1.15)	5.94 (5.08–6.94)	4.80 (2.36–9.75)	16.96 (14.38–20.01)
	Adjusted HR	1.03 (0.85–1.25)	2.42 (1.82–3.23)	Reference	2.09 (1.76–2.48)	0.88 (0.69–1.11)	3.42 (2.91–4.03)	3.66 (1.80–7.43)	6.20 (5.20–7.39)
Intra-abdominal	Crude HR	0.88 (0.75–1.05)	1.24 (0.90–1.72)	Reference	1.04 (0.88–1.23)	0.91 (0.74–1.11)	1.41 (1.21–1.64)	1.34 (0.43–4.17)	2.43 (1.98–2.98)
	Adjusted HR	1.01 (0.85–1.20)	1.26 (0.90–1.75)	Reference	0.93 (0.78–1.11)	0.87 (0.71–1.06)	1.03 (0.87–1.21)	1.10 (0.35–3.43)	1.29 (1.03–1.61)
Reproductive and urinary tract	Crude HR	1.30 (1.17–1.45)	1.80 (1.49–2.18)	Reference	1.46 (1.31–1.62)	0.95 (0.82–1.08)	2.21 (2.00–2.44)	4.19 (2.68–6.55)	5.75 (5.13–6.44)
	Adjusted HR	1.17 (1.05–1.31)	1.24 (1.02–1.50)	Reference	0.98 (0.88–1.10)	0.99 (0.86–1.14)	1.44 (1.30–1.60)	3.46 (2.21–5.41)	2.65 (2.34–3.01)
Skin and soft tissue	Crude HR	1.04 (0.84–1.30)	1.59 (1.07–2.35)	Reference	2.11 (1.73–2.58)	1.25 (0.97–1.60)	2.97 (2.47–3.58)	2.37 (0.75–7.44)	7.17 (5.80–8.86)
	Adjusted HR	1.19 (0.95–1.48)	1.36 (0.91–2.02)	Reference	1.58 (1.28–1.95)	1.17 (0.91–1.50)	1.75 (1.43–2.13)	1.82 (0.58–5.73)	2.68 (2.13–3.38)
Infection-related deaths	Crude HR	0.84 (0.19–3.75)	8.57 (1.92–38.29)	Reference	7.49 (2.30–24.42)	0.89 (0.15–5.32)	36.78 (11.77–114.92)	31.59 (3.29–303.69)	131.60 (41.87–413.65)
	Adjusted HR	0.96 (0.21–4.30)	8.47 (1.88–38.10)	Reference	5.99 (1.82–19.68)	0.87 (0.14–5.18)	20.42 (6.47–64.40)	23.65 (2.46–227.68)	46.09 (14.44–147.11)

*Adjusted HR for sex, BMI category, smoking, alcohol consumption, education level, diabetes (no, fasting glucose ≤130, 131–200, >200), systemic steroids use >30 days before study entry, and history of hospitalization within 6 months before hospitalization for infection syndrome.

For the risk of hospitalization for all infections, the P value for test for interaction <0.001. ^+^IncludingIncluding influenza, bacterial and viral pneumonia, bronchopneumonia and empyema.

### Site-specific infection

Older subjects had significantly higher aHRs for all site-specific infections, except only for intra-abdominal and reproductive and urinary tract infection in CKD stage 1. Younger patients with CKD stage 3 had increased risk except for intra-abdominal and skin and soft tissue infection. Moreover, although CKD stage 1 high patients also had higher risk for infection-related hospitalization, the sites differed in age strata. Despite their high eGFR, older CKD stage 1 high patients had increased aHR for all infections except for intra-abdominal and skin and soft tissue infection as if they had impaired renal function. Younger CKD stage 1 high patients suffered from only a slight but significant increase in risk of RUTI but no other types of infection (Table 4).

### Infection-related mortality

There were 402 infection-related deaths during the study period, with crude infection-related mortality rates per 1,000 person-years of 0.04 (95% CI: 0.02–0.07), 0.11 (95% CI: 0.08–0.15), 0.61 (95% CI: 0.54–0.70) and 2.87 (95% CI: 2.41–3.42) among participants with CKD stage 1 high, stage 1, stage 2 and 3, respectively. The aHR of infection-related mortality compared against those with CKD stage 1 was 1.39 (95%CI: 0.94–2.06) and 2.03 (95% CI: 1.32–3.12) for those with CKD stages 2 and 3, respectively. Compared with younger CKD stage 1 patients, their older counterparts had higher aHR of mortality in all CKD stages, including those with stage 1 high (aHR 8.47, 95% CI 1.88–38.10) (Table 4). Younger subjects showed no increase in aHR for infection-related mortality except for CKD stage 3 patients, whose aHR for infection related mortality was 23.65 (95% CI 2.46–227.68).

### Influence of proteinuria on risks of infection across different eGFR levels

**Figure 1:**
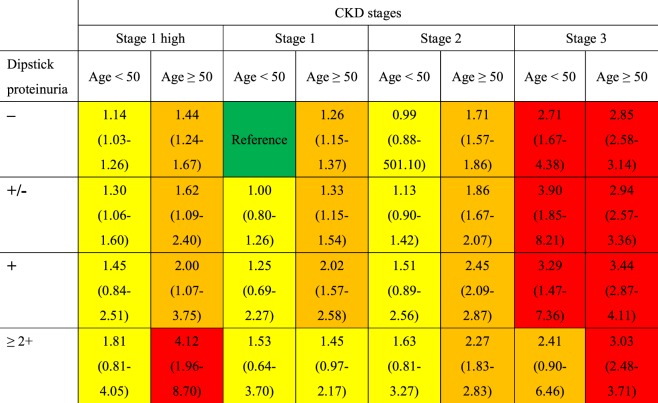
Adjusted hazard ration of hospitalization for all infections by estimated glomerular filtration rate (eGFR) and dipstick proteinuria categories. Adjusted for age category, sex, BMI category, smoking, alcohol consumption, education level, diabetes (no, fasting glucose ≤130, 131–200, >200), systemic steroids use >30 days before study entry, and history of hospitalization within 6 months before hospitalization for infection. Green, low risk; yellow, moderately increased risk; orange: high risk; red, very high risk.

Proteinuria posed additional risk of infection-associated hospitalization among CKD stage 3 patients, younger and older alike. However, among patients with milder CKD, proteinuria increased risk of infection-associated hospitalization only in older subjects (Fig. 1). In fact, older patients with CKD stage 1 high and proteinuria of 2+ had the highest risk (aHR 4.12, 95% CI 1.96–8.70), but this phenomenon was not observed among the younger subjects.

## Discussion

This study is not only one of the largest cohorts in the world but also the largest in Asia^7,16–19,21^. Previous studies were carried out in western countries with limited information regarding Asian subjects. Some of these studies enrolled mostly the elderly with an average age around 65 years^7,16,17^, or limited to certain conditions like diabetes mellitus^19^. The mean age of our subjects was 51.5 years with 45.5% of them younger than 50 years. Hence, this study can elucidate the impact of CKD on infection-associated hospitalization and mortality in a large, relatively young and healthy population. Current results indicated that participants aged <50 years with CKD stage 3 had significantly higher risks of infection-related hospitalization and mortality compared with their CKD stage 1 counterparts, and this risk is actually equal to that in older CKD patients aged ≥50 years. Younger CKD patients might have suboptimal therapeutic compliance for almost all key aspects of disease management, including treatment for diabetes, hyperlipidemia, and hypertension^22–25^ and even dialysis schedules, resulting in higher morbidity and mortality^26^. On the other hand, awareness and efforts against infection in younger CKD patients are far from adequate. For instance, Yu *et al*. showed that among Taiwanese diabetic patients, younger ones were less likely to receive influenza vaccination, and CKD patients were less likely to adhere to vaccination recommendations than people with other comorbidities such as congestive heart failure and chronic obstructive pulmonary disease^27^. Influenza and pneumococcal vaccinations are covered by public health services in Taiwan only for people aged above 65 and 75, respectively. For younger subjects, the vaccines will only be paid by the government if they reach ESRD. The present findings revealed an obvious gap between the need for prevention and actual prevention of infection in CKD patients; for younger patients with advanced CKD, prompt prevention, recognition, and treatment of infection is definitely necessary to reduce complications and save lives.

Proteinuria is not only a hallmark of CKD but also a valuable predictive factor for associated complications, including cardiovascular events; even trace proteinuria was associated with adverse outcomes, such as metabolic syndrome^28^. Reviewing the Atherosclerosis Risk in Communities Study (ARIC) database, Ishigami *et al*. pointed out that albuminuria is associated with infection-related hospitalization independent of the GFR level^17^. In our study, the risk of infection-related hospitalization started rising even with trace (+/−) dipstick proteinuria but only in older patients. Patients with a trace dipstick proteinuria are currently categorized as “normal to mildly increased (A1)”, according to the KDIGO 2012 Clinical Practice Guideline for the Evaluation and Management of Chronic Kidney Disease, and assigned to routine observation only^29,30^; despite that their actual risk of infection is already increasing. Although dipstick urinalysis might be less accurate than the urine albumin-to-creatinine ratio, the present findings demonstrated its usefulness in predicting risks of infection-associated hospitalization and mortality in older CKD patients. Even more striking is that, in older CKD patients, those with CKD stage 1 high and heavy proteinuria had the highest risk of infection-related hospitalization, even higher than their CKD stage 3 counterparts. The current results highlighted that even in patients with normal eGFR level, proteinuria is a red flag of infection-related morbidity, especially in older patients. Aggressive nephrologist referral and investigation of not only the causes of proteinuria but also predisposing factors for infection in these subgroups are indispensable to improving patient-care quality.

The strength of this study is the large number of participants from a community health screening program and the prospective long follow-up period. Hospitalization for infection was documented by linkage to the National Health Insurance (NHI) Database, capturing both community- and hospital-acquired infections with a very low missing rate^31^. However, this study also has some limitations. First, categorizing participants according to a single measurement of eGFR can result in misclassification. However, the misclassification bias regarding the eGFR level is considered non-differential. Second, the prevalence of CKD in our study population is lower than the national prevalence, which might result from selection bias of a health screening program, with only a small number of advanced CKD cases. Third, CKD patients may have admission rate bias;for example physicians may more likely be admitting CKD patients than those with normal renal function. However, the fact that younger patients were admitted as frequently as older ones speaks against selection bias, at least in groups with advanced CKD. Fourth, although risk factors were promptly adjusted, unmeasured confounding variables such as duration of diabetes or socioeconomic status might exist. Fifth, underdiagnoses of some sites of infection, such as intra-abdominal infection, is possible since imaging studies with contrast medium are limited in advanced CKD patients. The high mortality rate in older CKD patients may lead to overestimation of infection-related hospitalizations with some of them dead before being admitted. If death is treated as a competing risk for infection-related hospitalization, the risk of infection-related hospitalization in the younger CKD stage 3 patients might exceed that in their older counterparts. Finally, the present findings have their basis on observations in a relatively homogenous ethnic background with fewer comorbid conditions; therefore, generalization to other patient populations requires further evaluation

## Conclusion

Younger CKD patients are at high risk of infection-related hospitalization and mortality. Moreover, identification of proteinuria is suboptimal especially in those with high eGFR levels, who actually have as high risk as those with low eGFR levels. Despite the current lack of effective prevention against infection in CKD patients, adequate vaccination and aggressive recognition and treatment of modifiable conditions such as proteinuria are mandatory.

## Materials and Methods

### Data sources and study population

A total of 125,865 individuals who participated voluntarily in a free community-based health screening service for residents aged 20 years or older in New Taipei City from 2005 to 2008 were the potential subjects for this prospective study. The details of this study had been described elsewhere^32^. In brief, the participants filled out a questionnaire on their demographics, educational level, and lifestyle. Each participant received a standard physical examination including anthropomorphic measurements as well as blood and urine analyses. Overnight fasting blood and first morning spot urine were collected and analyzed. Individual identifications were removed and the subjects became anonymous after enrollment. The screening program database was linked to the NHI Database and the National Death Registry using each participant’s unique national identification number. In Taiwan, national health insurance is compulsory for all residents, and the coverage rate is >99%. The protocol was approved by the National Taiwan University Hospital Research Ethics Committee^32^. All participants or their legal guardians provided written consent. The study was carried out in accordance with the good clinical research practice guidelines and regulations

Participants were excluded if they (1) did not have a baseline measurement of serum creatinine, body mass index (BMI), and fasting glucose level; (2) did not have complete information about cigarette smoking, alcohol consumption, and education level; (3) did not have any claims in the NHI Database; and (4) received a kidney transplantation, hemodialysis, or peritoneal dialysis therapy during the entire study period^32^.

### Measurements

The main measurement of this study was baseline renal function, calculated using the CKD-EPI equation^33^. Participants were classified as follows: CKD stage 1 high, >105 ml/min/1.73 m^2^; CKD stage 1, 90–105 ml/min/1.73 m^2^; CKD stage 2, 60–89 ml/min/1.73 m^2^; and CKD stage 3, 59–30 ml/min/1.73 m^2^. Dipstick proteinuria was detected by urinalysis of a single random spot urine. Diabetes was defined by a fasting plasma glucose (FPG) exceeding 126 mg/dL or a claim for more than 28 days of hypoglycemic agent in the health insurance databasewithin one year before the baseline survey. The body mass index (BMI) was the quotient of weight (in kilograms) and the square of height (in meters). Weight and height were self-reported. The BMI was categorized as follows: underweight, <18.5 kg/m^2^; normal, ≥18.5 kg/m^2^ and <25 kg/m^2^; overweight, ≥25 kg/m^2^ and <30 kg/m^2^; and obese, ≥30 kg/m^2^. The baseline demography and behavioral risk factors such as smoking and alcohol consumption were obtained from the questionnaire. Comorbidity, systemic steroid use for >30 days in the year prior to study entry, and hospitalization within 6 months before index infection-related hospitalization were retrieved from the NHI Database. This study used codes from the International Classification of Diseases, 9^th^ revision, Clinical Modification [ICD-9-CM]^34^ (Supplementary Table 1) to ascertain the patients’ histories of diabetes mellitus, hypertension, cardiovascular disease, cerebrovascular disease, peripheral vascular disease, dyslipidemia, chronic liver and lung disease, autoimmune disease, and cancer. Information about renal replacement therapy was also obtained from the NHI Database.

### Outcomes and follow-up

The primary outcome was the first hospitalization for any infection ascertained from the NHI Database after enrollment. Hospitalizations were classified by the specific site of infection, including septicemia and bacteremia, lower respiratory tract (LRI) (including influenza, pneumonia and empyema), intra-abdominal, reproductive and urinary tract (RUTI), skin and soft tissue as defined by the ICD-9-CM codes listed in Supplementary Table 1. Codes had been extensively applied for various types of infection in the NHI Claims Database^31,35–41^. More than one specific site of infection can contribute to the first infection-related hospitalization. Recurrent infection-related hospitalizations were not analyzed because previous studies noted that the inclusion of recurrent episodes did not change the results much^17^; or could even skew the results favoring the survivors^40^. As for infection-related mortality, the vital status and date of death were ascertained with the National Death Registry by the participant’s unique identification number.

The participants were followed from the health screening date till the first infection-related hospitalization, death, or Dec 31, 2014, whichever came first. As for site-specific infection, participants hospitalized for one site of infection were not contributing follow-up person-time for another type of infection.

### Statistical analysis

The baseline characteristics of participants in different eGFR categories were compared. The crude incidence rates of overall and site-specific infection-related hospitalization and infection-related mortality were computed for the different eGFR groups. The adjusted hazard ratios (aHRs) and 95% confidence intervals (95% CIs) for infection-related hospitalizations and mortality for the different CKD categories were estimated using Cox regression modeling with CKD stage 1 patients as reference. Potential confounders, including age, sex, smoking, alcohol consumption, low educational level, diabetes (no diabetes, FPG ≤ 130, 131–200, and >200 mg/dL), systemic steroid use and hospitalization history within 6 months prior to index infection-related hospitalization were controlled for. Sensitivity analysis was conducted to ensure that the results were the same (Supplementary Table 2a–c). Subgroup analysis was also performed to examine whether the risks were modified by diabetes mellitus, age (<50 and ≥50 years), sex, and their serum albumin levels (≤4.0 and >4.0 g/dL). Cross-product terms were created and added to the multivariable Cox model; models with and without cross-product interaction terms were then compared using the likelihood ratio test. All analyses were conducted using SAS software version 9.4 (SAS Institute, Cary, North Carolina).

### Ethical approval

All procedures performed in this study were in accordance with the ethical standards of the National Taiwan University Hospital Research Ethics Committee (201805131 W) and with the 1964 Helsinki declaration and its later amendments or comparable ethical standards.

### Informed consent

Informed consent was obtained from all individual participants included in the study.

## Supplementary information

Supplementary Information.

## Data Availability

The dataset of the current study is available from the corresponding author on reasonable request.
