# Supplementary material for: Stratified risks of infection-related hospitalization in patients with chronic kidney disease - A prospective cohort study
Source: Sci Rep. 2020 Mar 11;10:4475. doi: 10.1038/s41598-020-61144-6 (PMC7066158; doi:10.1038/s41598-020-61144-6)
Supplement: Supplementary file 1 — Supplementary Information. [file 41598_2020_61144_MOESM1_ESM.docx]

**Title:**

**Stratified risks of infection-related hospitalization in patients with chronic kidney disease - A prospective cohort study**

**Authors:**

Wei-Shun Yang ^1,3^, Yi-Cheng Chang ^2,3,4,5^, Meng-Lun Hsieh ^2,4^, Jiun-Ling Wang* ^6,7^

Li-Chiu Wu ^4^, Chia-Hsuin Chang^4^,

**Supplementary Figure 1.** Flow chart for study participants’ enrollment.

| People participated in the New Taipei City Health Screening in 2005-2008 (N = 125,865) | |  |  |
| --- | --- | --- | --- |
|  |  |  |  |
|  |  | Excluded due to:  Age<20 years (N = 473)  Those who did not have baseline measurement of serum creatinine, BMI, and fasting glucose level (N = 1,853)  Those who did not have complete information about cigarette smoking, alcohol consumption, and education level (N = 2,438)  Those who did not have any claims in the National Health Insurance database (N = 242)  Those with eGFR < 30ml/min/1.72m^2^ (N = 299)  Those who received dialysis therapy or kidney transplantation (N = 689) | |
|  |  |  |  |
|  |  |  |  |
| Participants included in the analysis (N = 119,871)  eGFR > 105 (N = 28,013)  eGFR 90-105 (N = 40,502)  eGFR 60-89 (N = 45,665)  eGFR 30-59 (N = 5,691)) | |  |  |

**Supplementary Table 1. International Classification of Diseases, 9th revision, Clinical Modification (ICD-9-CM) codes used in the study**

| **Infection syndrome** |  |
| --- | --- |
| Septicemia | 038, 041.9, 790.7, 785.52 |
| Lower respiratory tract infection | 480, 487, 481, 482, 483, 485, 486, 510, 513 |
| Intra-abdominal infection | 540, 541, 542, 562.01, 562.03, 562.11, 562.13, 566, 567, 569.5, 572.0, 572.1, 575.0 |
| Reproductive and urinary tract infection | 590, 599.0, 601, 604, 614, 615, 616 |
| Skin and soft tissue infection | 680, 681, 682, 683, 684, 685, 686 |
| Osteomyelitis | 711.0, 730 |
| Necrotizing fasciitis | 728.86 |
| **Comorbidities** |  |
| Liver cirrhosis | 571.2, 571.5, 571.6 |
| Dialysis | ICD-9-CM: V451, V560, V568  Order code: 58001C, 58001CA, 58002C, 58002CB, 58009B, 58010A, 58010B, 58011A, 58011AB, 58011B, 58011C, 58012A, 58012B, 58013C, 58014C, 58017B, 58017C, 58018C, 58019C, 58020C, 58021C, 58022C, 58023C, 58024C, 58025C, 58026C, 58027C, 58028C, 58029C, 58030B, 69006C, 69006C, CGDW1960012C, CGS01440042C, CGS01440132C, CKD006752L2X, CKD006753L2X, CKDD1135132C, CKDD1170052C, CKDD117005KD, CKDD1170092C, CKDD117009KD, CKDD122334BQ, CKDD124334BQ, CKDD1390032C, CKDD139003KD, CKDD154334BQ, CKDD1881CK2C, CKDD1881SK2C, CKDD1883CK2C, CKDD1883SK2C, CKDD210598SB, CKDD2140012C, CKDD214001KD, CKDD2140022C, CKDD214002KD, CKDD214554NL, CKDD2330742C, CKDD2420052C, CKDD242005KD, CKDD2450072C, CKDD245007KD, CKDD245540SB, CKDD245542SB, CKDD2480012C, CKDD257331BA, CKDD2690012C, CKDD269001KD, CKDD269033BA, CKDD2881452C, CKDD288155KD, CKDD2SL12PM0, CKDD2SL18PM0, CKDD323334BQ, CKDD3456032C, CKDD345603KD, CKP010T2022C, CKP018812N2C, CKP018812NKD, CKP018814N2C, CKP018814NKD, CKP018817N2C, CKP018817NKD, CKP018880N2C, CKP018880NKD, CKP018884N2C, CKP018888N2C, CKP018890N2C, CKP018890NKD, CKP022C410BT, CKP0413836FA, CKP04C4407BT, CKP04C4479BT, CKP04C8303BT, CPC0260048DV, FUK0500M60GA, FUK050M100GA, FUK05ST100GA, FUK05ST60PGA, FUK05TS404FN, FUK05TS413FN, HEF03PRFPEGA, HEF03PRLPEGA, HEF03PRTPEGA |
| Hypertension | 401-404 |
| Ischemic heart disease | 411, 413, 414 |
| Myocardial infarction | 410, 412 |
| Cardiac dysrhythmia/atrial fibrillation | 427 |
| Congestive heart failure | 428, 398.91, 402.01, 402.11, 402.91, 404.01, 404.11, 404.91, 404.03, 404.13, 404.93 |
| Stroke | 430, 431, 432, 433, 434, 436 |
| Peripheral vascular disease | 440.2, 440.4, 443.81, 443.9 |
| Disorders of lipid metabolism | 272 |
| Chronic lung disease | 490-496, 500-508 |
| Autoimmune disease | 710.2, 696.0, 696.1, 714.0, 710.0, 710.1, 710.4, 283.0, 245.2, 340, 358.0, 364.0, 364.3, 555, 556 |
| Dementia | 290.0-290.4, 291.2, 294.1, 331.0-331.2, 290.10-290.13, 290.20, 290.21, 290.40-290.43, 294.10, 294.11, 331.11, 331.19, 331.82 |
| Cancer | 140-208 |
| Peptic ulcer disease | 531-535, 578.0, 578.1, 578.9 |
| **Medications** |  |
| Anti-diabetes medications | A10 |
| Systemic steroids | H02AA02, H02AB02, H02AB04, H02AB06, H02AB10, H02BX, H02BX91 |

**Sensitivity analyses and sub-group analysis**

Because infection will cause acute kidney injury, we excluded participants who were hospitalized for infection within two weeks after the enrollment. Next, we excluded those with the following diagnoses within one year prior to enrollment: a) systemic autoimmune disease, b) organ-specific autoimmune disease, c) malignant neoplasm of the bladder, kidney, and other unspecified urinary organs, d) obstructive uropathy, e) urinary stone, and f) hyperplasia of the prostate to see if the results changed substantially. Then, we shortened the maximal follow-up period to three years after the baseline to avoid a long time lag between the measurement of the baseline eGFR and infection outcome. We controlled for the influence of glycemic level by including the following variables in the multivariable model: no diabetes and diabetes with a FPG <90, 91-130, 131-200, and >200 mg/dL. Finally, in the multivariable regression model, we additionally adjusted for Charlson’s comorbidity score and dipstick proteinuria to see if the results would change substantially.

After excluding the possibility of reverse causation and confounding by other diseases that may potentially influence both renal function and infection risk, the relationship remained between the eGFR level and risk of hospitalized infection (Supplementary Table 2a-c). Diabetic participants with eGFR levels of 60-89 or 30-59 mL/min/1.73 m^2^ had increased risks of hospitalization for any infection and reproductive and urinary tract infections as compared with non-diabetic participants; although the difference was not statistically significant (Supplementary Table 3; *p* value for test for interaction = 0.10). Risks did not differ substantially between men and women, and those with normal and low serum albumin levels (Supplementary Tables 4 and 5).

**Supplementary Table 2a. Sensitivity Analysis 1 and 2**

|  | eGFR>105 | | *eGFR 60-89* | | *eGFR 30-59* | |
| --- | --- | --- | --- | --- | --- | --- |
|  | Sensitivity analysis 1 | Sensitivity analysis 2 | Sensitivity analysis 1 | Sensitivity analysis 2 | Sensitivity analysis 1 | Sensitivity analysis 2 |
| **Hospitalization for infection** |  |  |  |  |  |  |
| All infections | 1.18 (1.11~1.26) | 1.19 (1.11~1.27) | 1.05 (1.01~1.10) | 1.06 (1.01~1.11) | 1.42 (1.33~1.51) | 1.46 (1.36~1.57) |
| Septicemia | 0.99 (0.83~1.19) | 1.04 (0.86~1.27) | 1.05 (0.94~1.16) | 1.04 (0.93~1.17) | 1.41 (1.22~1.63) | 1.43 (1.21~1.68) |
| Lower respiratory tract | 1.24 (1.06~1.44) | 1.22 (1.03~1.44) | 1.06 (0.97~1.16) | 1.05 (0.95~1.16) | 1.41 (1.25~1.58) | 1.43 (1.25~1.63) |
| Intra-abdominal | 1.09 (0.93~1.26) | 1.06 (0.91~1.24) | 0.95 (0.85~1.07) | 0.93 (0.82~1.06) | 1.05 (0.85~1.29) | 1.03 (0.81~1.30) |
| Reproductive and urinary tract | 1.25 (1.14~1.37) | 1.26 (1.14~1.40) | 1.13 (1.06~1.21) | 1.15 (1.06~1.25) | 1.71 (1.55~1.90) | 1.87 (1.67~2.11) |
| Skin and soft tissue | 1.11 (0.93~1.32) | 1.13 (0.94~1.37) | 0.99 (0.88~1.11) | 1.01 (0.89~1.15) | 1.28 (1.08~1.53) | 1.25 (1.02~1.53) |
| **Infection-related deaths** | 1.62 (0.65~4.04) | 1.34 (0.51~3.53) | 1.39 (0.94~2.05) | 1.19 (0.78~1.82) | 2.04 (1.33~3.13) | 1.91 (1.19~3.07) |

*Adjusted for age category, sex, BMI category, smoking, alcohol consumption, education level, diabetes (no, fasting glucose ≤130, 131-200, >200), systemic steroids use >30 days before study entry, and history of hospitalization within 6 months before hospitalization for infection syndrome

Sensitivity analysis 1: Excluding those were hospitalized for infection within two weeks after health screening program (N=119,821)

Sensitivity analysis 2: Excluding those who had the following disease diagnoses within one year prior to health screening program: a) systemic autoimmune disease, b) organ specific autoimmune disease, c) malignant neoplasm of bladder, kidney, and other unspecified urinary organs, d) obstructive uropathy, e) urinary stone, and f) hyperplasia of prostate (N=105,110)

**Supplementary Table 2b. Sensitivity analysis 3 and 4**

|  | eGFR>105 | | *eGFR 60-89* | | *eGFR 30-59* | |
| --- | --- | --- | --- | --- | --- | --- |
|  | Sensitivity analysis 3 | Sensitivity analysis 4 | Sensitivity analysis 3 | Sensitivity analysis 4 | Sensitivity analysis 3 | Sensitivity analysis 4 |
| **Hospitalization for infection** |  |  |  |  |  |  |
| All infections | 1.17 (1.05~1.30) | 1.18 (1.11~1.26) | 1.01 (0.94~1.09) | 1.05 (1.01~1.10) | 1.42 (1.27~1.58) | 1.42 (1.33~1.51) |
| Septicemia | 0.86 (0.60~1.23) | 0.99 (0.83~1.19) | 0.98 (0.81~1.20) | 1.05 (0.94~1.16) | 1.43 (1.11~1.85) | 1.41 (1.22~1.62) |
| Lower respiratory tract | 1.44 (1.08~1.91) | 1.23 (1.06~1.44) | 1.07 (0.90~1.27) | 1.06 (0.97~1.16) | 1.45 (1.18~1.79) | 1.41 (1.25~1.58) |
| Intra-abdominal | 1.01 (0.79~1.29) | 1.08 (0.93~1.26) | 0.77 (0.64~0.94) | 0.96 (0.85~1.07) | 1.00 (0.73~1.38) | 1.06 (0.86~1.30) |
| Reproductive and urinary tract | 1.28 (1.10~1.49) | 1.25 (1.14~1.36) | 1.10 (0.97~1.24) | 1.14 (1.06~1.22) | 1.80 (1.52~2.14) | 1.73 (1.56~1.92) |
| Skin and soft tissue | 0.97 (0.73~1.28) | 1.10 (0.93~1.32) | 0.94 (0.77~1.14) | 0.98 (0.87~1.11) | 1.24 (0.94~1.65) | 1.28 (1.07~1.52) |
| **Infection-related deaths** | 5.26 (0.68~40.56) | 1.62 (0.65~4.04) | 1.23 (0.43~3.50) | 1.39 (0.94~2.05) | 1.52 (0.48~4.77) | 2.01 (1.31~3.09) |

Sensitivity analysis 3: Shortened the maximal follow-up period to three years after the baseline

Sensitivity analysis 4: Adjusted for age category, sex, BMI category, smoking, alcohol consumption, education level, diabetes (no, fasting glucose <90, 91-130, 131-200, >200), systemic steroids use >30 days before study entry, and history of hospitalization within 6 months before hospitalization for infection syndrome

**Supplementary Table 2c. Sensitivity analysis 5**

|  | eGFR>105 | *eGFR 60-89* | *eGFR 30-59* |
| --- | --- | --- | --- |
| **Hospitalization for infection** |  |  |  |
| All infections | 1.18 (1.11~1.26) | 1.05 (1.00~1.10) | 1.37 (1.28~1.46) |
| Septicemia | 0.99 (0.83~1.19) | 1.04 (0.94~1.16) | 1.36 (1.17~1.57) |
| Lower respiratory tract | 1.23 (1.06~1.44) | 1.05 (0.96~1.15) | 1.35 (1.20~1.52) |
| Intra-abdominal | 1.08 (0.93~1.26) | 0.95 (0.85~1.07) | 1.04 (0.84~1.27) |
| Reproductive and urinary tract | 1.25 (1.14~1.37) | 1.13 (1.05~1.21) | 1.67 (1.51~1.85) |
| Skin and soft tissue | 1.11 (0.93~1.32) | 0.98 (0.87~1.10) | 1.23 (1.03~1.46) |
| **Infection-related deaths** | 1.63 (0.65~4.06) | 1.39 (0.94~2.05) | 1.98 (1.29~3.04) |

Sensitivity analysis 5: Adjusted for age category, sex, BMI category, smoking, alcohol consumption, education level, diabetes (no, fasting glucose <90, 91-130, 131-200, >200), Charlson’s comorbidity score, systemic steroids use >30 days before study entry, and history of hospitalization within 6 months before hospitalization for infection syndrome

**Supplementary Table 3. Subgroup analysis: adjusted hazard ratios for different eGFR categories and risk of hospitalization for infection syndrome and infection-related mortality as compared with eGFR eGFR 90-105 in participants with diabetes (N = 10,424) and those without diabetes (N = 109,447)**

|  | eGFR>105 | | *eGFR 60-89* | | *eGFR 30-59* | |
| --- | --- | --- | --- | --- | --- | --- |
|  | With diabetes* | Without diabetes† | With diabetes* | Without diabetes† | With diabetes* | Without diabetes† |
| **Hospitalization for infection** |  |  |  |  |  |  |
| All infections | 1.13 (0.94~1.37) | 1.17 (1.09~1.25) | 1.18 (1.06~1.31) | 1.03 (0.98~1.08) | 1.64 (1.44~1.88) | 1.38 (1.28~1.48) |
| Septicemia and bacteremia | 1.09 (0.71~1.68) | 0.95 (0.78~1.16) | 1.09 (0.88~1.36) | 1.03 (0.91~1.16) | 1.54 (1.17~2.02) | 1.37 (1.16~1.63) |
| Lower respiratory tract^+^ | 1.09 (0.72~1.64) | 1.23 (1.04~1.45) | 1.11 (0.91~1.36) | 1.05 (0.95~1.16) | 1.45 (1.13~1.85) | 1.42 (1.24~1.62) |
| Intra-abdominal | 0.93 (0.56~1.56) | 1.09 (0.93~1.28) | 0.94 (0.68~1.30) | 0.96 (0.84~1.08) | 1.06 (0.67~1.69) | 1.07 (0.85~1.35) |
| Reproductive and urinary tract | 1.28 (0.95~1.73) | 1.23 (1.12~1.36) | 1.42 (1.20~1.67) | 1.08 (1.00~1.17) | 2.22 (1.81~2.72) | 1.61 (1.43~1.82) |
| Skin and soft tissue | 0.98 (0.62~1.57) | 1.10 (0.91~1.33) | 1.00 (0.78~1.29) | 0.98 (0.86~1.12) | 1.33 (0.95~1.86) | 1.30 (1.06~1.59) |
| **Infection-related deaths** | 1.03 (0.18~6.01) | 1.77 (0.60~5.20) | 1.59 (0.80~3.15) | 1.35 (0.84~2.18) | 2.31 (1.08~4.94) | 1.98 (1.18~3.33) |

*Adjusted for age category, sex, BMI category, smoking, alcohol consumption, education level, fasting glucose (≤130, 131-200, >200), systemic steroids use >30 days before study entry, and history of hospitalization within 6 months before hospitalization for infection syndrome

†Adjusted for age category, sex, BMI category, smoking, alcohol consumption, education level, systemic steroids use >30 days before study entry, and history of hospitalization within 6 months before hospitalization for infection syndrome

For the risk of hospitalization for all infections, the P value for test for interaction = 0.10

+ Including influenza, bacterial and viral pneumonia, bronchopneumonia and empyema.

**Supplementary Table 4. Subgroup analysis: adjusted hazard ratios for different eGFR categories and risk of hospitalization for infection syndrome and infection-related mortality as compared with eGFR eGFR 90-105 in men (N = 42,762) and women (N = 77,109)**

|  | eGFR>105 | | *eGFR 60-89* | | *eGFR 30-59* | | *eGFR<30 but not on dialysis therapy* | |
| --- | --- | --- | --- | --- | --- | --- | --- | --- |
|  | Men | Women | Men | Women | Men | Women | Men | Women |
| **Hospitalization for infection** |  |  |  |  |  |  |  |  |
| All infections | 1.26 (1.10~1.43) | 1.07 (1.00~1.16) | 0.99 (0.92~1.07) | 1.07 (1.01~1.13) | 1.25 (1.13~1.38) | 1.53 (1.40~1.67) | 2.04 (1.60~2.61) | 2.56 (2.09~3.14) |
| Septicemia | 0.96 (0.68~1.35) | 1.01 (0.81~1.26) | 0.91 (0.76~1.08) | 1.13 (0.99~1.30) | 1.22 (0.98~1.52) | 1.50 (1.22~1.83) | 1.65 (0.94~2.89) | 2.36 (1.48~3.78) |
| Lower respiratory tract | 1.36 (1.03~1.78) | 1.07 (0.88~1.29) | 0.93 (0.81~1.06) | 1.16 (1.02~1.31) | 1.14 (0.97~1.34) | 1.73 (1.45~2.06) | 1.69 (1.15~2.47) | 1.94 (1.22~3.09) |
| Intra-abdominal | 1.35 (1.07~1.72) | 1.02 (0.84~1.24) | 0.93 (0.79~1.11) | 1.00 (0.85~1.17) | 1.11 (0.84~1.47) | 1.00 (0.73~1.37) | 0.61 (0.15~2.47) | 1.96 (0.91~4.25) |
| Reproductive and urinary tract | 1.31 (0.99~1.74) | 1.13 (1.02~1.24) | 1.11 (0.95~1.29) | 1.12 (1.03~1.21) | 1.44 (1.18~1.75) | 1.80 (1.59~2.04) | 2.45 (1.53~3.92) | 2.97 (2.25~3.93) |
| Skin and soft tissue | 1.16 (0.87~1.55) | 1.10 (0.88~1.39) | 0.96 (0.80~1.15) | 1.01 (0.86~1.18) | 1.30 (1.02~1.67) | 1.26 (0.97~1.62) | 3.28 (1.87~5.74) | 2.46 (1.41~4.32) |
| **Infection-related deaths** | 3.76 (1.24~11.40) | 0.62 (0.12~3.31) | 0.97 (0.57~1.64) | 1.81 (1.02~3.21) | 1.33 (0.75~2.36) | 2.85 (1.49~5.43) | 0.83 (0.19~3.69) | 6.45 (2.56~16.23) |

*Adjusted for age category, BMI category, smoking, alcohol consumption, education level, diabetes (no, fasting glucose ≤130, 131-200, >200), systemic steroids use >30 days before study entry, and history of hospitalization within 6 months before hospitalization for infection syndrome

For the risk of hospitalization for all infections, the P value for test for interaction = 0.10

**Supplementary Table 5. Subgroup analysis: adjusted hazard ratios for different eGFR categories and risk of hospitalization for infection syndrome and infection-related mortality as compared with eGFR eGFR 90-105 in participants with serum albumin level ≤ 4.0 (N = 5,216) and those with serum albumin level > 4.0 (N = 114,655**)

|  | eGFR>105 | | *eGFR 60-89* | | *eGFR 30-59* | | *eGFR<30 but not on dialysis therapy* | |
| --- | --- | --- | --- | --- | --- | --- | --- | --- |
|  | Serum albumin ≤ 4.0 | Serum albumin >4.0 | Serum albumin ≤ 4.0 | Serum albumin >4.0 | Serum albumin ≤ 4.0 | Serum albumin >4.0 | Serum albumin ≤ 4.0 | Serum albumin >4.0 |
| **Hospitalization for infection** |  |  |  |  |  |  |  |  |
| All infections | 0.93 (0.65~1.33) | 1.17 (1.10~1.25) | 1.00 (0.80~1.25) | 1.06 (1.01~1.11) | 1.55 (1.19~2.01) | 1.40 (1.31~1.50) | 2.73 (1.79~4.16) | 2.33 (1.96~2.78) |
| Septicemia | 0.90 (0.42~1.95) | 1.00 (0.83~1.21) | 1.11 (0.70~1.76) | 1.04 (0.93~1.16) | 1.82 (1.07~3.11) | 1.35 (1.16~1.58) | 2.66 (1.08~6.53) | 1.96 (1.31~2.93) |
| Lower respiratory tract | 0.96 (0.43~2.14) | 1.23 (1.05~1.44) | 1.19 (0.77~1.85) | 1.06 (0.96~1.16) | 2.13 (1.31~3.46) | 1.37 (1.21~1.54) | 1.98 (0.83~4.67) | 1.93 (1.41~2.65) |
| Intra-abdominal | 0.85 (0.38~1.91) | 1.07 (0.92~1.25) | 0.61 (0.33~1.11) | 0.98 (0.87~1.10) | 1.27 (0.57~2.84) | 1.05 (0.85~1.30) | 3.62 (1.04~12.63) | 0.88 (0.36~2.13) |
| Reproductive and urinary tract | 1.12 (0.65~1.95) | 1.23 (1.12~1.35) | 1.16 (0.80~1.68) | 1.13 (1.05~1.21) | 2.11 (1.37~3.26) | 1.69 (1.52~1.88) | 4.41 (2.28~8.55) | 2.84 (2.18~3.70) |
| Skin and soft tissue | 0.97 (0.43~2.19) | 1.09 (0.90~1.31) | 0.75 (0.42~1.34) | 1.00 (0.88~1.12) | 1.36 (0.68~2.73) | 1.27 (1.06~1.52) | 2.20 (0.65~7.39) | 2.88 (1.88~4.40) |
| **Infection-related deaths** | 1.33 (0.10~17.08) | 1.60 (0.60~4.27) | 1.29 (0.34~4.85) | 1.42 (0.95~2.14) | 2.41 (0.60~9.78) | 2.00 (1.27~3.14) | 1.43 (0.13~16.32) | 3.76 (1.73~8.16) |

*Adjusted for age category, BMI category, smoking, alcohol consumption, education level, diabetes (no, fasting glucose ≤130, 131-200, >200), systemic steroids use >30 days before study entry, and history of hospitalization within 6 months before hospitalization for infection syndrome

For the risk of hospitalization for all infections, the P value for test for interaction = 0.2
